# Supplementary material for: Macrofungi Cultivation in Shady Forest Areas Significantly Increases Microbiome Diversity, Abundance and Functional Capacity in Soil Furrows
Source: J Fungi (Basel). 2021 Sep 18;7(9):775. doi: 10.3390/jof7090775 (PMC8469386; doi:10.3390/jof7090775)
Supplement: Supplementary file 1 [file jof-07-00775-s001.zip › Fig. S1_ new.pdf]

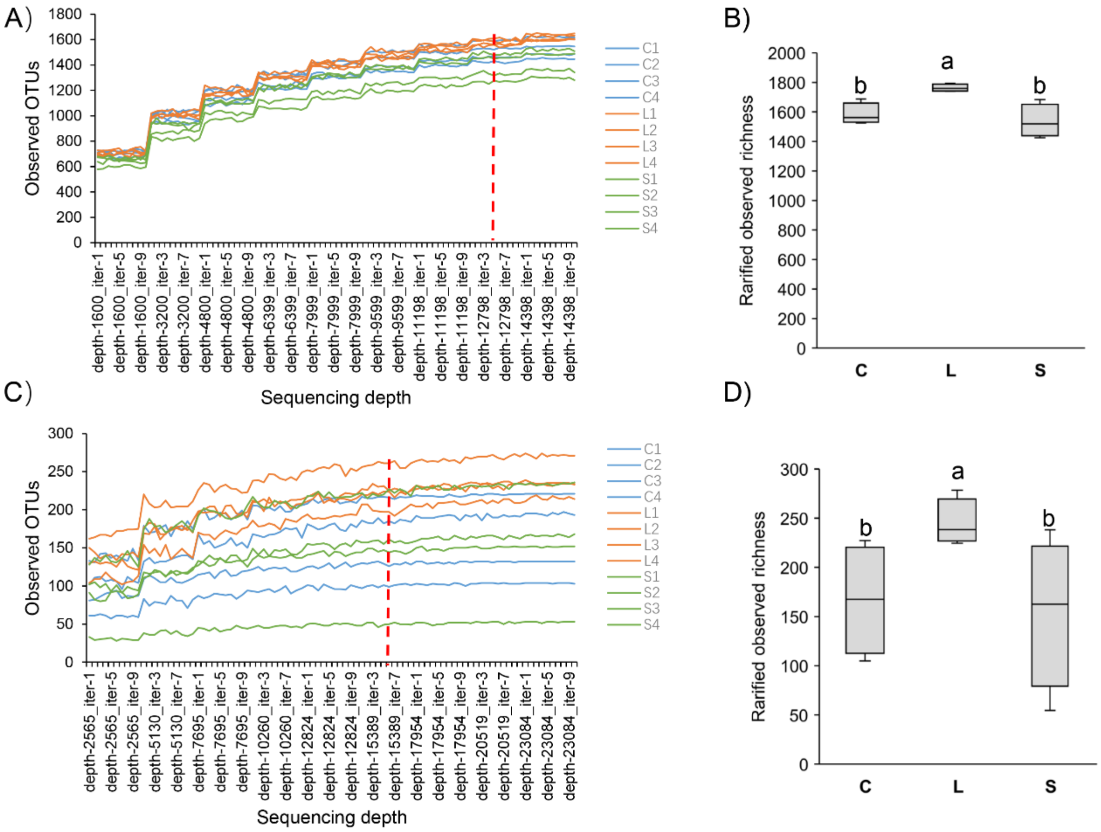

Figure S1. Rarefaction curves for bacteria (**A, B**) and fungi (**C, D**) observed OTUs richness. The dashed red line indicates the selected rarefaction sequencing depth used to generate the box plots of rarified observed richness: 12,798 and 15,389 sequences per sample for bacterial and fungal communities, respectively. C =, Control forest soil with no influence from mushroom cultivation; L = Soil forest from furrows with low influence by mushroom cultivation; S = Forest soil below fungal beds strongly influenced by mushroom cultivation. Different letters indicate significant differences at p = 0.05 (ANOVA) between means (Tukey's HSD pairwise comparisons, n = 4).
