# Supplementary material for: Macrofungi Cultivation in Shady Forest Areas Significantly Increases Microbiome Diversity, Abundance and Functional Capacity in Soil Furrows
Source: J Fungi (Basel). 2021 Sep 18;7(9):775. doi: 10.3390/jof7090775 (PMC8469386; doi:10.3390/jof7090775)
Supplement: Supplementary file 1 [file jof-07-00775-s001.zip › Supplementary file 1-proof.pdf]

Macrofungi Cultivation in Shady Forest Areas Significantly Increases Microbiome Diversity, Abundance and Functional Capacity in Soil Furrows

Dong Liu [1], Yanliang Wang [1], Peng Zhang [1], Fuqiang Yu [1,*] and Jesús Perez-Moreno [2*]

SUPPLEMENTARY METHODS

Soil chemical analyses

Soil moisture was measured gravimetrically by oven drying moist soil samples at 105 °C for 24 h. pH was determined in a soil to water mixture of dry soil and distilled water (1:2.5, *w*/*v*). Soil organic matter (OM) content was determined with the potassium dichromate external heating method [1]. Soil organic carbon (SOC) was determined via wet oxidation using dichromate in an acid medium followed by the $FeSO_4$ titration method [2]. Total nitrogen (TN) contents were measured with Kjeldahl digestion and distillation azotometry following the extraction with 0.02 mol L^{-1} sulfuric acid [3]. Total phosphorus (TP) was measured spectrophotometrically after wet digestion with H_2SO_4 and $HClO_4$ followed by colorimetric analysis (UV 2800) [4]. These nutrient concentrations were used to calculate soil C:N, C:P and N:P mass ratios. The alkaline hydrolysable nitrogen (AN) content was determined using the alkaline-hydrolysable diffusion method [2]. The available phosphorus (AP) was extracted with sodium bicarbonate and determined using the molybdenum-blue method [5].

The available manganese (Mn) and iron (Fe) in soil samples were determined by inductively coupled plasma mass spectrometry with diethylenetriaminepentaacetic (DTPA) acid extraction as described by Nu (1999). The ion chromatography was used to determine the content of calcium (Ca^{2+}), and magnesium ions (Mg^{2+}) based on the national standard method NY-T 296-1995. Soil samples were dissolved in hydrochloric acid, followed by the separation by cation ions exchange chromatography column, and then the cation suppressor was used for the precision, repeatability, recovery, system suitability tests, and the final results test respectively.

Soil acid phosphatase, hydrolases (glucosidase and cellobiosidase) and oxidase activities were measured photometrically. Soil acid phosphatase (S-ACP) activity assay kit (Solarbio® Life Science, Beijing, China) was used for S-ACP activity. 0.1 g fresh soil (2 mm screened) was dissolved in 0.05 mL methylbenzene, with a general mixture for 15 min. Then the chemicals were added step by step following the kit instructions. In acidic environment, S-ACP catalyzes the hydrolysis of benzene disodium phosphate to phenol and disodium hydrogen phosphate. The activity of S-ACP was calculated by measuring the amount of phenol produced. For other enzyme activities, 1 g of fresh soil was dissolved in 125 mL 50 mM sodium acetate buffer (pH = 5). Then, the suspension was prepared by stirring with magnetic stirrers for 1 min. After that, the steps were followed according to the instructions for soil α-glucosidase (S-α-GC) and β-1, 4-cellobiosidase (S-C1) and polyphenoloxidase (S-PPO) activity assay kits. Final processed solutions were determined by colorimetric method at light absorption at 400 nm (S-α-GC and S-C1) and 430 nm (S-PPO), respectively.

SUPPLEMENTARY REFERENCES

1. Guo, M. Soil Sampling and Methods of Analysis. *J. Environ. Qual.* **2009**, *38*, 375, doi:10.2134/jeq2008.0018br.
2. Bao, S.D. Soil agricultural chemistry analysis, 3rd edition.; China Agriculture Press: Beijing, China, **2007**, 265–267.
3. Sparks, D.L.; Page, A.L.; Helmke, P.A.; Loeppert, R.H. *Methods of Soil Analysis Part 3—Chemical Methods*; SSSA Book Series; Soil Science Society of America, American Society of Agronomy: Madison, WI, USA, **1996**; ISBN 978-0-89118-866-7**.**
4. Parkinson, J.A.; Allen, S.E. A wet oxidation procedure suitable for the determination of nitrogen and mineral nutrients in biological material. *Commun. Soil Sci. Plant Anal.* **1975**, *6*, 1–11, doi:10.1080/00103627509366539.
5. 5. 5. Nu, R.K. Soil agricultural chemical analysis; China Agricultural Science and Technology Press, 1999.
